# Relationship between clinical features and the diagnostic value of CMR tissue characterisation in patients with clinically suspected myocarditis

**DOI:** 10.1186/1532-429X-17-S1-P375

**Published:** 2015-02-03

**Authors:** Stefan P Biesbroek, Esha Kumar, Aernout M Beek, Albert C van Rossum

**Affiliations:** 1 Cardiology, VU University Medical Center, Amsterdam, Netherlands

## Background

Myocarditis can present with acute coronary syndrome-like (ACS) symptoms, new-onset heart failure (NOHF) or life threatening arrhythmias (LTA) with various ECG - and laboratory test abnormalities. Cardiac magnetic resonance (CMR) tissue characterization using Late Gadolinium Enhancement (LGE) and T2 weighted (T2W) imaging is the non-invasive diagnostic tool of choice. The relationship between the clinical features of myocarditis and the diagnostic value of CMR however is unclear.

## Methods

All patients admitted with clinically suspected myocarditis according to recent guidelines, were retrospectively included. All patients routinely underwent T2W and LGE imaging within three weeks of admission. CMR diagnosis of myocarditis required typical midwall or subepicardial contrast enhancement. Patient were classified into acute or non-acute myocarditis based on T2W images.

## Results

82 patients were included in the present study. Using CMR imaging, myocarditis was diagnosed in 49% of the patients, of whom 83% were classified as acute and 13% as non-acute. Other pathology was diagnosed in 16% of the cases (myocardial infarction 11%, dilated cardiomyopathy 2%, takotsubo cardiomyopathy 2%). One patient had myocardial edema on T2W images without LGE. T2W images were non-interpretable in 5 patients because of insufficient image quality. In 2 patients LGE findings were inconclusive. In the remaining 25 patients (30%), CMR imaging did not reveal myocardial tissue abnormalities. CMR diagnosis of myocarditis significantly varied with the type of clinical presentation and was higher in ACS (61%, n=31/51) than in in NOHF (37%, n=3/11) and LTA (30%, n=6/20) presentations. Patients with negative CMR studies less often had elevated CK-MB or elevated CRP (87% vs. 71%, p=0.120; 67% vs. 33%, p=0.026 respectively). Patients with CMR myocarditis had higher CRP, CK-MB and troponin T than patients with normal tissue characteristics. (59.2±80.8 vs. 18.9±38.1, p=0.004; 38.5±39.1 vs. 12.1±11.6, p=0.001; 0.98±1.09 vs. 0.34±0.47, p=0.004 respectively). CK-MB was furthermore significantly correlated with the extent of LGE, expressed as percentage of total myocardial mass (Pearson's r=0.55, p < 0.001).

## Conclusions

CMR using tissue characterization diagnoses myocarditis only in the minority of patients with suspected disease and mainly so in those with ACS presentation or significant enzyme release. Our findings stress the importance of the use of newer mapping techniques in these patients for a more detailed tissue characterization.

## Funding

None.

